# Extracellular Vesicles Derived From Murine Cementoblasts Possess the Potential to Increase Receptor Activator of Nuclear Factor-κB Ligand-Induced Osteoclastogenesis

**DOI:** 10.3389/fphys.2022.825596

**Published:** 2022-02-14

**Authors:** Rei Sato, Kentaro Maruyama, Eiji Nemoto, Yukihiko Sakisaka, Shigeki Suzuki, Jiajun Li, Kento Numazaki, Hiroyuki Tada, Satoru Yamada

**Affiliations:** ^1^Division of Periodontology and Endodontology, Tohoku University Graduate School of Dentistry, Sendai, Japan; ^2^Division of Orthodontics and Dentofacial Orthopedics, Tohoku University Graduate School of Dentistry, Sendai, Japan; ^3^Division of Oral Immunology, Tohoku University Graduate School of Dentistry, Sendai, Japan

**Keywords:** extracellular vesicles, cementoblasts, RANKL, osteoclastogenesis, cementum resorption

## Abstract

Cementum resorption, unlike bone resorption, is clinically known to occur only with limited pathological stimuli, such as trauma, orthodontic forces, and large apical periodontitis; however, the molecular mechanisms that control osteoclast formation on the cementum surface remain unclear. In this study, we focused on extracellular vesicles (EVs) secreted by cementoblasts and analyzed their effects on osteoclast differentiation. EVs were extracted from the conditioned medium (CM) of the mouse cementoblast cell line OCCM-30. Transmission electron microscopy (TEM) analysis confirmed the presence of EVs with a diameter of approximately 50–200 nm. The effect of the EVs on osteoclast differentiation was examined using the mouse osteoclast progenitor cell line RAW 264.7 with recombinant receptor activator of nuclear factor (NF)-κB ligand (rRANKL) stimulation. EVs enhanced the formation of tartrate-resistant acid phosphatase (TRAP) activity-positive cells upon rRANKL stimulation. EVs also enhanced the induction of osteoclast-associated gene and protein expression in this condition, as determined by real-time PCR and Western blotting, respectively. On the other hand, no enhancing effect of EVs was observed without rRANKL stimulation. A Western blot analysis revealed no expression of receptor activator of NF-κB ligand (RANKL) in EVs themselves. The effect on rRANKL-induced osteoclast differentiation was examined using the CM of cementoblasts in terms of TRAP activity-positive cell formation and osteoclast-associated gene expression. The conditioned medium partly inhibited rRANKL-induced osteoclast differentiation and almost completely suppressed its enhancing effect by EVs. These results indicate that cementoblasts secreted EVs, which enhanced RANKL-induced osteoclast differentiation, and simultaneously produced soluble factors that neutralized this enhancing effect of EVs, implicating this balance in the regulation of cementum absorption. A more detailed understanding of this crosstalk between cementoblasts and osteoclasts will contribute to the development of new therapies for pathological root resorption.

## Introduction

During bone remodeling, bone resorption by osteoclasts is followed by bone formation by osteoblasts. Osteoclast precursor cells, derived from monocyte/macrophage lineage, differentiate into large, multinucleated tartrate-resistant acid phosphatase (TRAP)-positive mature cells in response to receptor activator of nuclear factor (NF)-κB ligand (RANKL), which is essential for osteoclastogenesis (Jimi et al., 1999; Kong et al., 1999; Kitaura et al., 2020; Holliday et al., 2021). RANKL exerts its osteoclastogenic effect by binding with its receptor receptor activator of NF-κB (RANK), a type I membrane protein expressed by osteoclast precursors. The critical role of RANKL-RANK signaling in bone resorption has been demonstrated by numerous *in vitro* and *in vivo* studies (Jimi et al., 1999; Kong et al., 1999; Kitaura et al., 2020; Holliday et al., 2021). The RANKL-RANK pathway is inhibited by osteoprotegerin (OPG), a member of the tumor necrosis factor (TNF) receptor superfamily, which acts as a soluble decoy receptor for RANKL and competes for binding to RANKL with RANK, thus blocking RANKL-induced osteoclastogenesis (Boyle et al., 2003). The binding of RANKL to RANK on the surface of osteoclast precursor cells results in the recruitment of TNF receptor-associated factor 6 (TRAF6), which is involved in the activation of downstream signaling pathways, such as mitogen activated protein kinases (MAPKs) and NF-κB. This signaling activates various transcription factors, such as NF-κB, c-Fos, and nuclear factor-activated T cells c1 (NFATc1), which are responsible for osteoclast differentiation (Boyle et al., 2003). Particularly, NFATc1, a master regulator of osteoclast differentiation, directly regulates a number of osteoclast-associated genes coding for acid phosphatase 5 (ACP5, also known as TRAP), osteoclast-associated receptor (OSCAR), cathepsin K (CtsK), osteoclast stimulatory transmembrane protein (OC-STAMP), and dendritic cell specific transmembrane protein (DC-STAMP; Boyle et al., 2003; Matsumoto et al., 2004; Kim et al., 2005).

Unlike the significant bone resorption in inflammatory conditions in the oral cavity, especially in periodontitis, tooth root resorption is rarely seen. Cementum is a thin calcified tissue covering the root and is an important component for fixing the root and surrounding alveolar bone/connective tissue (Saygin et al., 2000; Bosshardt, 2005). Cementum shares many properties with bone and has a similar biochemical composition. Although it is unknown whether cementoblasts and osteoblasts have a common progenitor cell, cementum, unlike bone, lacks innervation and vascularization and has very limited remodeling capacity (Saygin et al., 2000; Bosshardt, 2005). Furthermore, cementum, as opposed to bone, is generally considered to be resistant to osteoclast resorption, but cementum resorption is often induced in lesions caused by pathological stimuli such as trauma, orthodontic forces, or large periapical periodontitis (Feller et al., 2016). However, little is known about the mechanisms that control osteoclast formation on the cementum surface.

Extracellular vesicles (EVs) are newly identified membrane vesicles with lipid bilayer structures and play important roles as regulators of tissue homeostasis. EVs are generally classified into two different classes, exosomes and microvesicles, mainly according to their size and biosynthetic mechanisms (Camussi et al., 2010). Exosomes have an endosome origin and are rather homogenous with diameters ranging from 30 to 200 nm. Microvesicles are membrane vesicles 100–1,000 nm in diameter that are secreted by budding from the plasma membrane. EVs are secreted by various cells and are present in most body fluids. EVs are enriched in bioactive molecules, such as proteins, lipids, and nucleic acids, including mRNA, microRNA (miRNA), and non-coding RNA, which are transferred between cells, thereby affecting the phenotype and function of the target cells (Camussi et al., 2010). Furthermore, the molecular composition of EVs varies not only with the cell type and origin, but also with the cell activation/differentiation status, even in the same parental cells (Ferguson and Nguyen, 2016). There is increasing evidence that EVs released from cells of each organ or tissue can function as a means of intercellular communication during physiological processes, such as cardiac remodeling (Barile et al., 2017), airway remodeling (Kulshreshtha et al., 2013; Haj-Salem et al., 2018), bone remodeling (Deng et al., 2015; Cui et al., 2016; Cappariello et al., 2018; Wang et al., 2018), tissue repair (Lamichhane et al., 2015; Anderson et al., 2016), and pathological processes, including pro-inflammation (Zhang et al., 2017; Kojima et al., 2018)/anti-inflammation (Jiang et al., 2019; Ni et al., 2019). In the last few years, EVs have been reported to control similar systems in periodontal tissues. EVs released from periodontal ligament cells in mechanical environments contributed to the maintenance of periodontal immune/inflammatory homeostasis (Wang et al., 2019); osteocyte-derived EVs induced by mechanical stretch forces promoted periodontal ligament cell proliferation and osteogenic differentiation (Lv et al., 2020); and gingival mesenchymal stem cell EVs facilitated M1 macrophage transformation into M2 macrophages (Wang et al., 2020; Nakao et al., 2021). These findings suggest the significant role of EVs in the maintenance of periodontal tissue homeostasis; however, there is currently no information on their contribution in cementum biology.

In this study, EVs were purified from a mouse cementoblast cell line, OCCM-30, and investigated for their role in osteoclastogenesis. We demonstrated that cementoblast-derived EVs have the potential to increase RANKL-induced osteoclast formation. This study is the first to report the biological activity of cementoblast-derived EVs and contributes to the elucidation of new crosstalk between cementoblasts and osteoclasts.

## Materials and Methods

### Reagents

Recombinant murine RANKL was purchased from PeproTech (Rocky Hill, NJ, United States). Recombinant human parathyroid hormone related protein (PTHrP 1-34) was purchased from FUJIFILM Wako Pure Chemical Corporation (Osaka, Japan). Cytochalasin D and Triton X-100 was purchased from Sigma-Aldrich (St. Louis, MO, United States). Exoquick-TC™ was purchased from System Biosciences LLC (Palo Alto, CA, United States). Hoechst 33342 was purchased from Immunochemistry Technologies (Bloomington, MN, United States).

### Cell Line and Cell Culture

OCCM-30, an immortalized murine cementoblast cell line (D’Errico et al., 2000), was maintained in Dulbecco’s Modified Eagle’s Medium (DMEM; Thermo Fisher Scientific, Waltham, MA, United States) or α-Minimum Essential Medium (α-MEM; Thermo Fisher Scientific) containing 10% heat-inactivated fetal bovine serum (FBS; Biowest, Nuaillé, France), 100 U/ml penicillin G, and 100 μg/ml streptomycin. RAW 264.7, a murine monocyte cell line, was obtained from KAC Corporation (Kyoto, Japan) and maintained in DMEM containing 10% FBS, 100 U/ml penicillin, and 100 μg/ml streptomycin.

### Preparation of Conditioned Medium and Isolation of EVs

Extracellular vesicles were isolated from cell conditioned medium (CM) using the Exoquick-TC™ (System Biosciences LLC, CA, United States) in accordance with the manufacturer’s protocol. Briefly, OCCM-30 cells were cultured in a 60-mm dish (5 ml/well) until confluent in DMEM with 5% FBS. Cells were then cultured for a further 48 h in medium containing 5% FBS from which EVs had been removed by the FBS Exosome Depletion Kit I-Column Format (Norgen Biotek, Thorold, ON, Canada), and culture supernatants were recovered. The cell pieces were removed by centrifugation at 1,500 rpm for 5 min, and the culture supernatant from which apoptotic bodies were removed was recovered by centrifugation at 3,000 × *g* for 30 min. About 2 mm of culture supernatant was added to the tube with Exoquick-TC™ solution and allowed to stand at 4°C overnight. The next day, the supernatant was centrifuged at 1,500 × *g* for 30 min, and pellets of the remaining EVs after removal of the supernatant were suspended in phosphate-buffered saline (PBS) and stored at −80°C. The protein concentration of EVs was measured using the Protein Assay BCA Kit (FUJIFILM Wako Pure Chemical Corporation). Theoretically, 20 μg of EVs was recovered from 1.0 ml of CM, which corresponds to 3 × 10^5^ OCCM-30 cells.

### Identification of EVs

The ultrastructure of the EVs was analyzed using transmission electron microscopy (TEM). Purified EVs were inspected using a HITACHI H-7600 at 100 kV (Hanaichi Ultrastructure Research Institute, Aichi, Japan). Approximately 5 μl of a sample (0.5 μg/μl) was placed on parafilm. A carbon-coated 400-mesh copper grid was then positioned on top of the drop for 10 s and washed with a droplet of distilled water. The grid was contrasted by adding a drop of 2% uranyl acetate to the parafilm and incubating the grid on the top of the drop for 10 s. Excess liquid was gently removed using absorbing paper. After drying, EVs were observed by TEM. The particle size and distribution of EVs were assessed by nanoparticle tracking analysis (NTA) by NanoSight LM 10 (Malvern Panalytical Ltd., Malvern, United Kingdom). The camera level was set at 13, and the image was photographed five times at 25 frames/second for 60 s. The particle size and particle density were calculated from the image analysis. The results were analyzed using NTA software (Nanoparticle Tracking Analysis Version 2.3 Build 0033) according to the manufacturer’s instructions.

### Uptake of EVs by Monocytes

Purified EVs were labeled with the PKH67 Green Fluorescent Cell Linker Kit® (Merck KGaA, Darmstadt, Germany) according to the manufacturer’s protocol. Briefly, 2 μl PKH67 dye was added into 250 μl Diluent C (Sigma-Aldrich). EVs in PBS were added into the PKH67 dye mixture at a volume of 3:1 and cultured at room temperature for 5 min. Unincorporated dye from labeled EVs preparations was removed by centrifugation using Exosome Spin Columns® (MW 3000; Thermo Fisher Scientific). The labeled EVs re-suspended in 20 μl PBS, which corresponded to 1 ml of 1:2 diluted original culture supernatant, were incubated with RAW 264.7 cells already seeded at 6 × 10^4^ cells/well (2 ml) on a 35-mm poly-L-lysine-coated glass-bottomed dish (Matsunami Glass Ltd., Osaka, Japan) for 6 and 24 h, and staining was evaluated by immunofluorescence microscopy. Nuclei were stained with Hoechst 33342 for 5 min.

### *In vitro* Osteoclast Formation Assay

RAW 264.7 cells were seeded into 24-well plates at a density of 1.5 × 10^4^ cells/well (0.5 ml), allowed to attach for 24 h, and then cultured in the presence or absence of 50 ng/ml recombinant murine RANKL in combination with 20 μg/ml EVs or 50% (v/v) CM for 5 days in α-MEM (0.5 ml/well) containing 10% FBS. At the end of the culture period, the cells were fixed in 100% methanol for 15 min, permeabilized with 0.1% Triton X-100 in PBS, and then stained for TRAP using a TRAP/ALP Stain Kit (FUJIFILM Wako Pure Chemical Corporation). TRAP-positive osteoclasts were quantified by counting the number of multinucleated red-stained cells (≥3 nuclei). Results are expressed as the number of TRAP-positive cells per well.

### Reverse Transcription and Real-Time Quantitative PCR

Total RNA was extracted using the Qiashredder and RNeasy® Kits (QIAGEN, Valencia, CA, United States) according to the manufacturer’s instructions and then treated with DNase (DNA-free™, Ambion Inc., Austin, TX, United States). The total RNA obtained was reverse transcribed (RT) at 25/10, 55/30, and 85/5 [temperature (°C)/time (s)] using a Transcriptor First Strand cDNA Synthesis Kit® (Roche Diagnostic Co., Indianapolis, United States) and iCycler® (Bio-Rad Laboratories, Hercules, CA, United States), and 50 ng of the transformed cDNA was used for the quantitative RT-PCR. In real-time PCR, the amplification profile was 40 cycles at 95/3, 60/20 [temperature (°C)/time (sec)]. PCR was performed using the CFX96 Touch™ Real-Time PCR Detection System (Bio-Rad Laboratories, Hercules, CA, United States) with a KAPA SYBR® FAST Kit (Kapa Biosystems, Cape Town, South Africa) and optimized levels of 3 mM MgCl_2_ and 500 nM of each primer. The relative expression levels of the transcripts are shown after normalization to the corresponding sample expression level of glyceraldehyde 3-phosphate dehydrogenase (GAPDH). The sequences of the primers for the murine genes encoding cathepsin K (Ctsk), Nfatc1, Oscar, Acp5, OC-STAMP, DC-STAMP, RANK, and GAPDH are as follows: *Ctsk* (5′-TAGCACCCTTAGTCTTCCGC-3'/5'-TTGAACACCCACATCCTGCT-3); *Oscar* (5'-TGCTGGTAACGGATCAGCTC-3'/5'-AACAGTAGGTGCCAGGTGTG-3'); *Nfatc1* (5'-CATGCGAGCCATCATCGAC-3'/5'-GGATGTGAACTCGGAAGACC3'); *Acp5* (5'-TGAACCATGAGAAGTATGACAACA-3'/5'-TATCTCCACATGTGTGAAGCCG3'); *Oc-stamp* (5'-TTCCTCTTCACCTGCTGTGC-3'/5'-CAGTGGAACAACTGCCTTGC3'); *Dc-stamp* (5'-CTTGTGGAGGAACCTAAGCGG-3'/5'-GGATGAAGTCCAGCCAGCTA3'); *Tnfrsf11a* (*Rank*; 5'-CTTGCAGCTCAACAAGGATACG-3'/5'-GGAAGAGCTGCAGACCACAT-3'); and *Gapdh* (5'-AATGTGTCCGTCGTGGATCTGA-3'/5'-GATGCCTGCTTCACCACCTTCT-3').

### Western Blotting

Cells were lysed with Cell Lysis Buffer® (Cell Signaling Technology, Beverly, MA, United States) according to the product manual. Whole cell lysates or purified EVs were treated with 2 × Laemmli sample buffer (Bio-Rad Laboratories), separated by sodium dodecyl sulfate-polyacrylamide gel electrophoresis, and transferred to a polyvinylidene difluoride membrane (ATTO, Tokyo, Japan) using a semidry transblot system (ATTO). The blot was blocked with 0.5% (w/v) non-fat dried milk and 0.1% (v/v) Tween 20 in PBS at room temperature for 1 h, followed by an incubation at room temperature for 1 h with rabbit anti-exosome flotillim-1 (Cell Signaling Technology) at 1:1,000, mouse anti-alix (Cell Signaling Technology) at 1:1,000, mouse anti-CD63 (Biolegend, San Diego, CA, United States) at 1:1,000, rabbit anti-calreticulin (Cell Signaling Technology) at 1:1,000, mouse anti-RANKL (Santa Cruz Biotechnology Inc., Dallas, Texas, United States) at 1:1,000, rabbit anti-β-actin (Cell Signaling Technology) at 1:1,000, rabbit anti-Ctsk (ProteinTech, Rosemont, IL, United States) at 1:1,000 or rabbit anti-TSG101 polyclonal (Novus Biologicals, LLC, Centennial, Colorado) at 1:500, and rabbit anti-NFATc1 (Biolegend) at 1:500. Blots were incubated with HRP-conjugated goat anti-rabbit IgG (Cell Signaling Technology) at 1:2,000, or HRP-conjugated goat anti-mouse IgG (Cell Signaling Technology) at 1:1,000 at room temperature for 1 h. Blots were then treated with Amersham™ ECL™ Prime Western Blotting Detection Reagents (Cytiva, Marlborough, MA, United States), and chemiluminescent signals were detected using an luminescent image analyzer (ChemiDoc XRS Plus™, Bio-Rad Laboratories).

### Statistical Analysis

All experiments were repeated three times to test the reproducibility of the results and representative results are shown as means ± SD. Differences between control and experimental groups were evaluated by the Kruskal-Wallis test with the Steel *post hoc* test. Values of *p* < 0.05 were regarded as significant.

## Results

### Characterization of Cementoblast-Derived EVs

**Figure 1:**
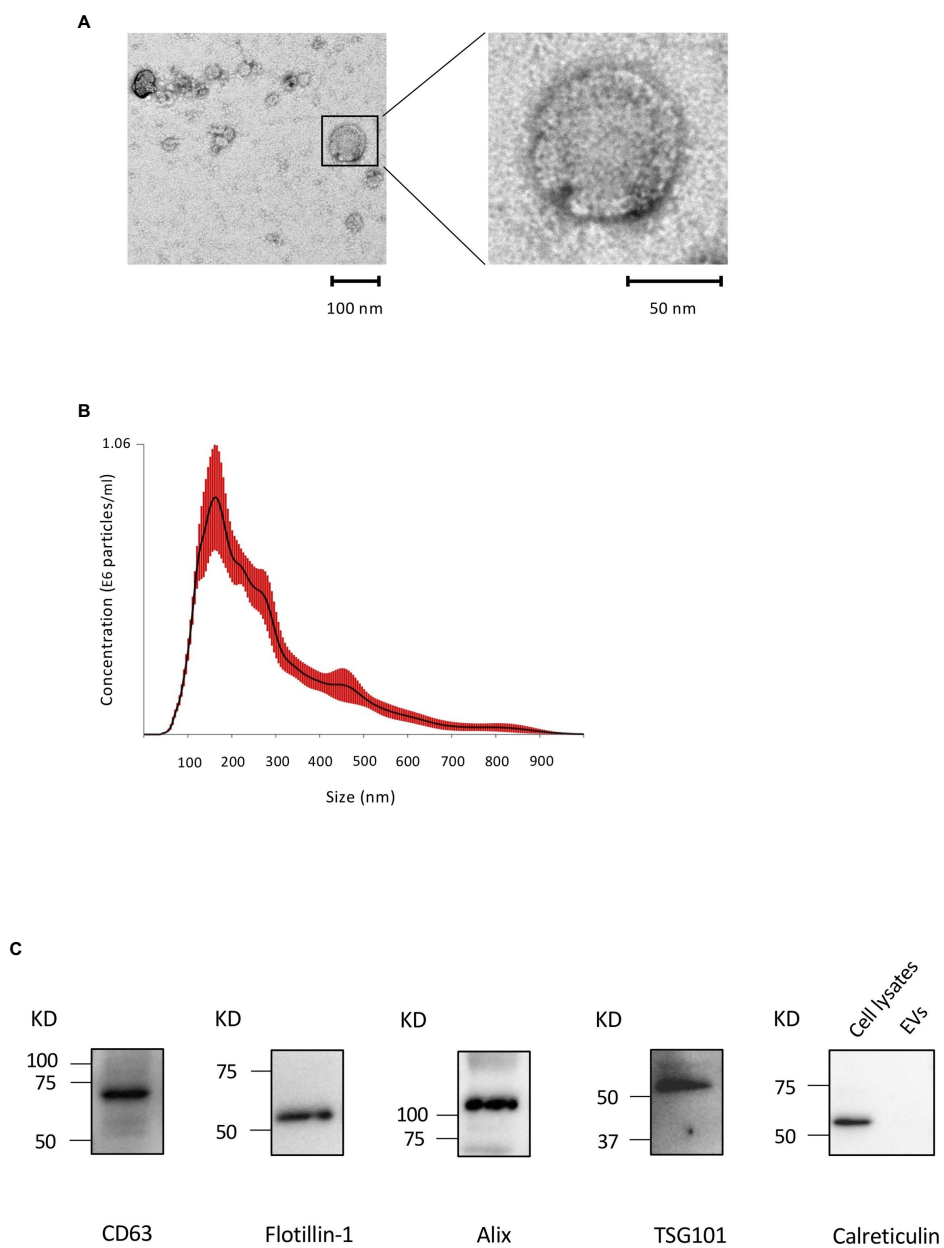
Characterization of cementoblast-derived extracellular vesicles (EVs). **(A)**: Morphology of EVs by transmission electron microscopy (TEM). **(B)**: Particle size and concentration of EVs was measured by nanoparticle tracking analysis (NTA). Red error bars indicate SE. **(C)**: Immunoblots of exosomal marker proteins (Flotillin-1, ALIX, CD63, and TSG101) in purified EVs and a non-exosomal marker (calreticulin) in cell lysates from OCCM-30 cells.

We first performed TEM to detect the existence of EVs from the CM of OCCM-30 cells. A typical EV shape was detected: classic cup-shaped, round morphology with diameters of approximately 100 nm (Figure 1A). The presence of EVs was further confirmed using NTA. Figure 1B shows the NAT profiles of EVs with a single peak and diameters ranging between 50 and 900 nm with a mode average of 181 ± 24.8 nm, which is consistent with the characteristic size range of EVs. The total theoretical concentration of EVs from 2 × 10^5^ cells was 1.92 ± 0.12 E^9^ particles in 1 ml of original CM. A Western blot analysis revealed the expression of several exosome-associated markers, such as Flotillin-1, Alix, CD63, and TSG101, were detected on the EVs, and endoplasmic reticulum-associated (cytoplasmic) marker, calreticulin (Rosenberger et al., 2019), was detected on the cell lysates but not on EVs (Figure 1C).

### Uptake of EVs by Monocytes

**Figure 2:**
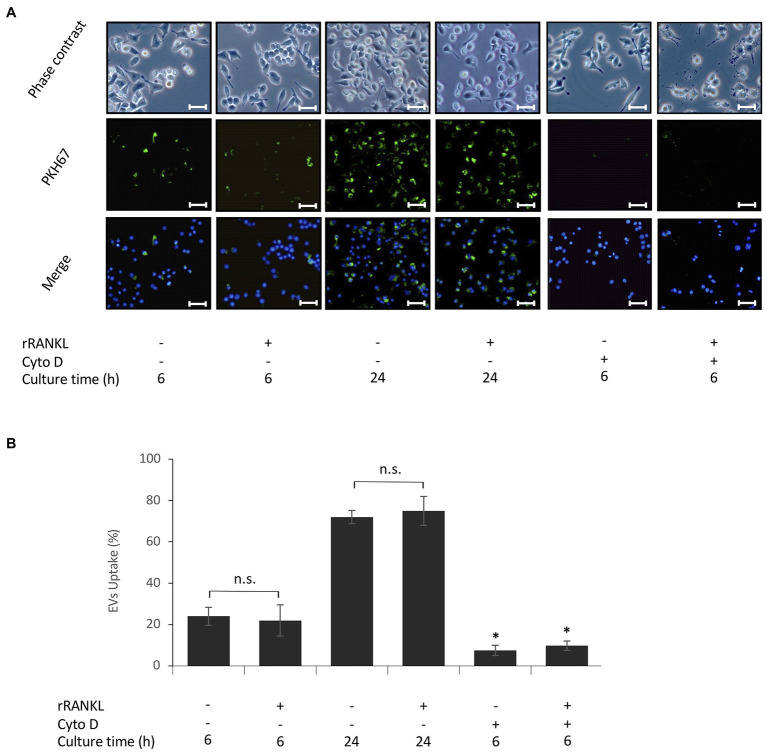
Uptake of EVs by monocytes. RAW 264.7 cells were incubated in the presence of 20 μg/ml PKH67-labeled EVs with or without 50 ng/ml recombinant receptor activator of nuclear factor (NF)-κB ligand (rRANKL) and with or without cytochalasin D (Cyto D) for 6 and 24 h. **(A)**: Phase contrast is shown in the upper panels. EVs taken up by RAW 264.7 cells (light green in middle panel) were detected by immunostaining after 6 and 24 h. Nuclei were visualized by staining with Hoechst 33342 (blue in lower panel), and merged images are shown in the lower panel (magnification: ×400, scale bars: 50 μm). **(B)**: The number of cells stained with PKH67-labeled EVs was counted in three randomly selected fields (each containing ~100 cells). Representative data from three separate experiments are shown as the means ± SD of triplicate assays (^*^p < 0.05 compared with the respective control; ns, not significant).

Next, we investigated whether cementoblast-derived EVs contribute to osteoclast formation using RAW 264.7 cells, which differentiate into osteoclasts upon rRANKL stimulation. First, to examine the uptake of EVs by RAW 264.7 cells, EVs were labeled with PKH67, a fluorescent dye that is incorporated into the lipid membrane of EVs, and co-incubated with the cells. An immunofluorescence microscopic analysis showed that approximately 22% of EVs was taken up by RAW 264.7 cells 6 h after the addition of the EVs and approximately 78% after 24 h (Figures 2A,B). EV uptake was not significantly affected by rRANKL stimulation (Figures 2A,B). It is known that actin polymerization is required for the uptake of EVs by macrophages (Sinha et al., 2016). To verify that EVs are internalized, but not simply attached to the surface of the cell membrane, RAW 264.7 cells were pretreated with cytochalasin D, an inhibitor of actin polymerization. As shown in Figures 2A,B, few cells showed fluorescence, indicating that EVs are indeed taken up by RAW 264.7 cells.

### EVs Enhance RANKL-Induced Osteoclast Formation

**Figure 3:**
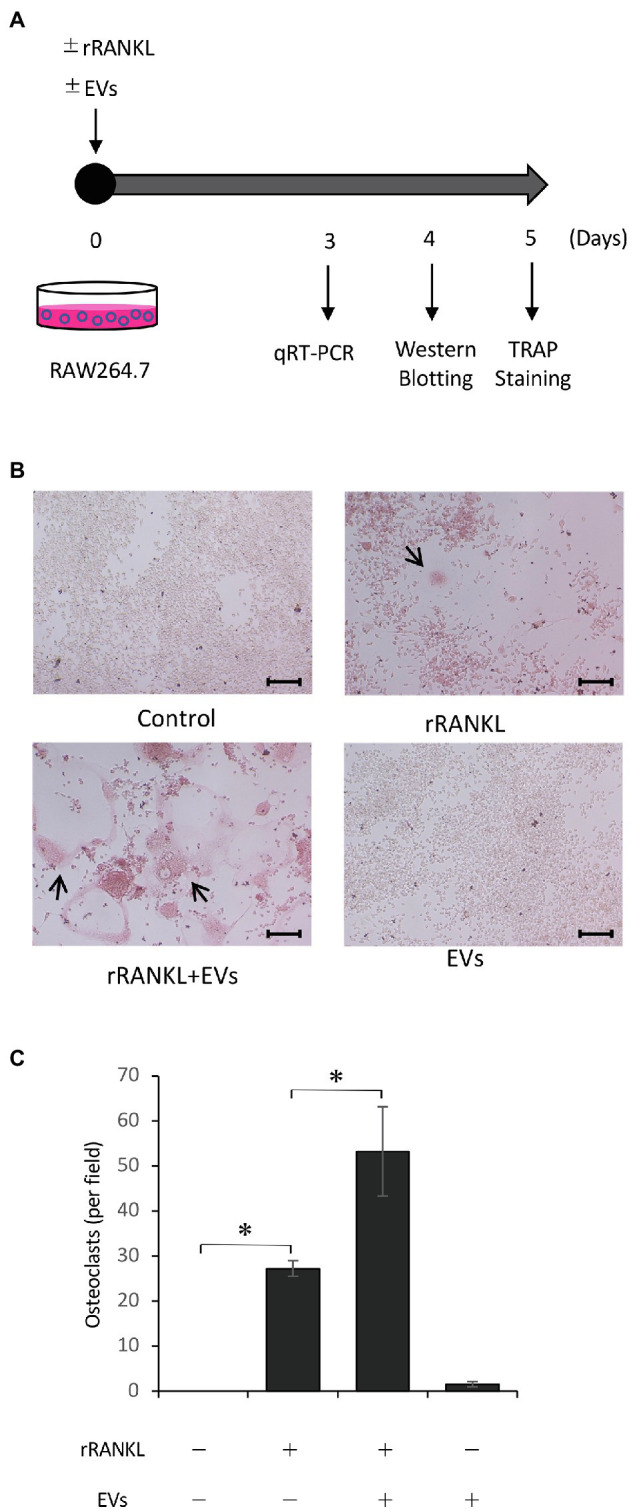
Cementoblast-derived EVs enhance receptor activator of NF-κB ligand (RANKL)-induced osteoclast formation. RAW 264.7 cells were stimulated with rRANKL (50 ng/ml) in the presence or absence of 20 μg/ml EVs derived from OCCM-30 cells for 5 days as illustrated in **(A)**. **(B)**: Tartrate-resistant acid phosphatase (TRAP) staining was performed. The TRAP-positive cells were marked with arrows (scale bars: 100 μm). **(C)**: Quantification of the TRAP-positive osteoclasts shown in **(B)**. Results are representative **(B)** or the mean ± SE **(C)** of at least three independent experiments (^*^p < 0.05 compared with the respective control).

To examine the effects of cementoblast-derived EVs on osteoclastogenesis, RAW 264.7 cells were cultured in the presence of 50 ng/ml rRANKL for 5 days and subjected to TRAP staining (as shown in Figure 3A). As shown in Figures 3B,C, rRANKL induced osteoclast differentiation with multinucleation and enhanced the intensity of TRAP staining. The addition of cementoblast-derived EVs to the rRANKL-treated group enhanced rRANKL induced-osteoclast differentiation, as shown by the increased number of multinucleated osteoclasts and higher intensity of TRAP staining. On the other hand, the stimulation with EVs alone did not induce multinucleated osteoclasts, and the intensity of TRAP staining was similar to that of the control. No significant effects were observed when RAW 264.7 cell-derived EVs were used as control EVs (Supplementary Figures 1A,B). These data suggest that cementoblast-derived EVs enhanced RANKL-induced osteoclast differentiation but not in the absence of rRANKL.

### EVs Enhance RANKL-Induced Osteoclast-Associated Molecules

**Figure 4:**
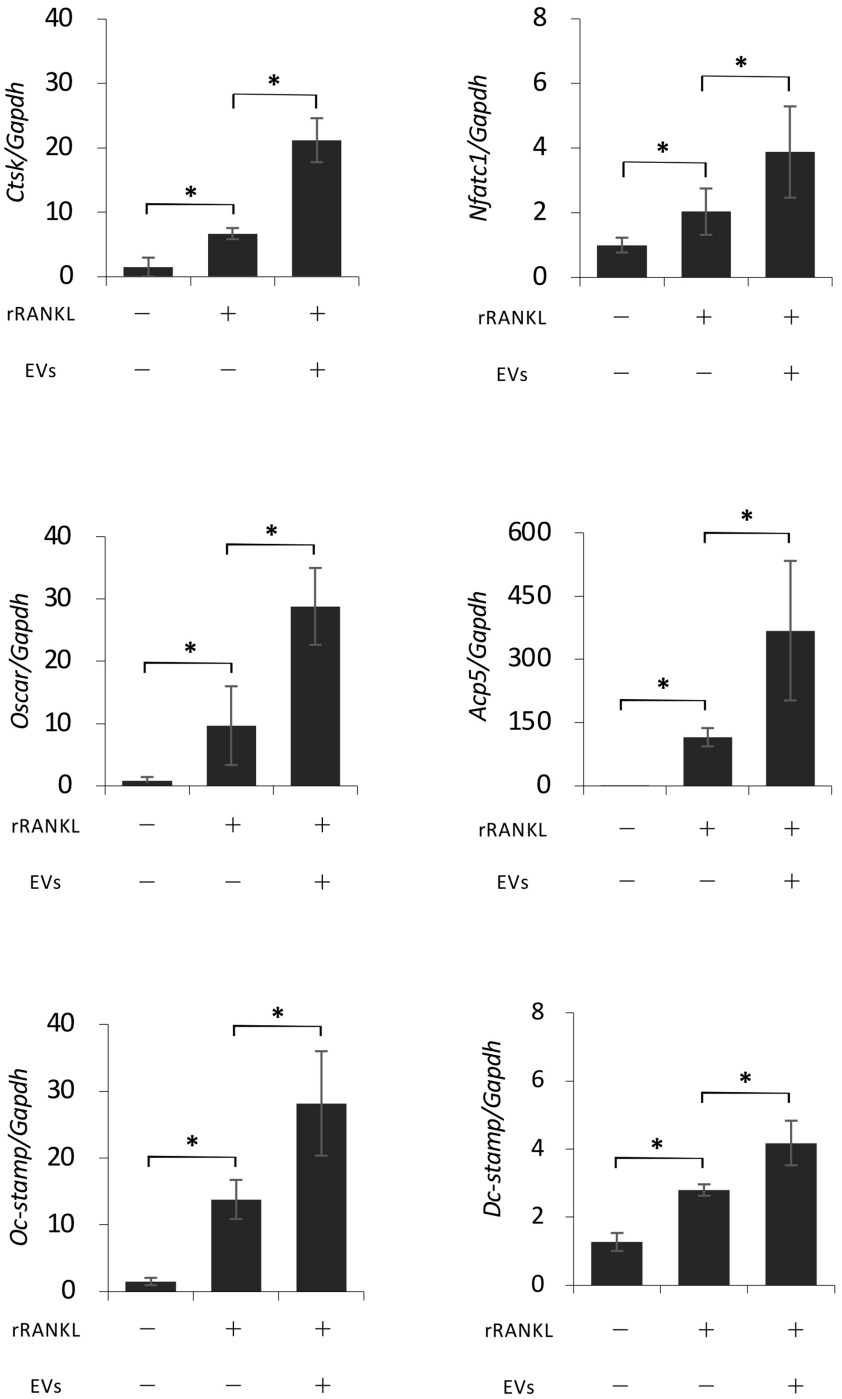
Extracellular vesicles enhance RANKL-induced osteoclast-associated molecules at the gene level. RAW 264.7 cells were stimulated with rRANKL (50 ng/ml) in the presence or absence of 20 μg/ml EVs derived from OCCM-30 cells for 3 days. Total cellular RNA was extracted, and transcripts of nuclear factor-activated T cells c1 (*Nfatc1*), cathepsin K (*Ctsk*), osteoclast-associated receptor (*Oscar*), acid phosphatase 5 (*Acp5*), *Dcstamp*, and *Ocstamp* were analyzed by real-time quantitative PCR. Representative findings of three independent experiments are shown (^*^p < 0.05 compared with the respective control).

**Figure 5:**
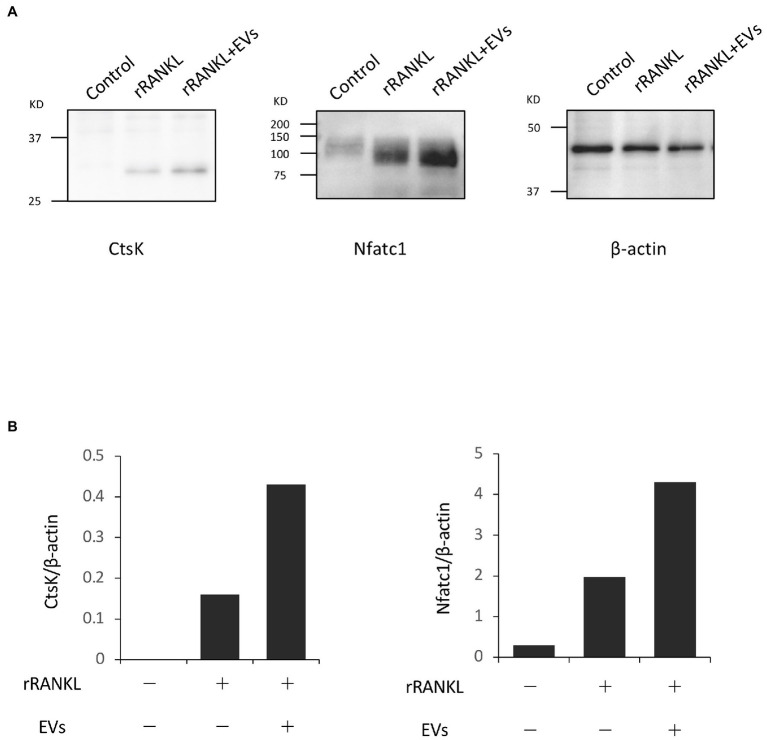
Extracellular vesicles enhance RANKL-induced osteoclast-associated molecules at the protein level. RAW 264.7 cells were stimulated with rRANKL (50 ng/ml) in the presence or absence of 20 μg/ml EVs derived from OCCM-30 cells for 4 days. Total cellular protein was extracted, and Western blotting was performed using antibodies for Nfatc1 and Ctsk. **(A)**: Expression of each protein. **(B)**: Each band was scanned with a densitometer and normalized to β-actin. Representative findings of three independent experiments are shown.

**Figure 6:**
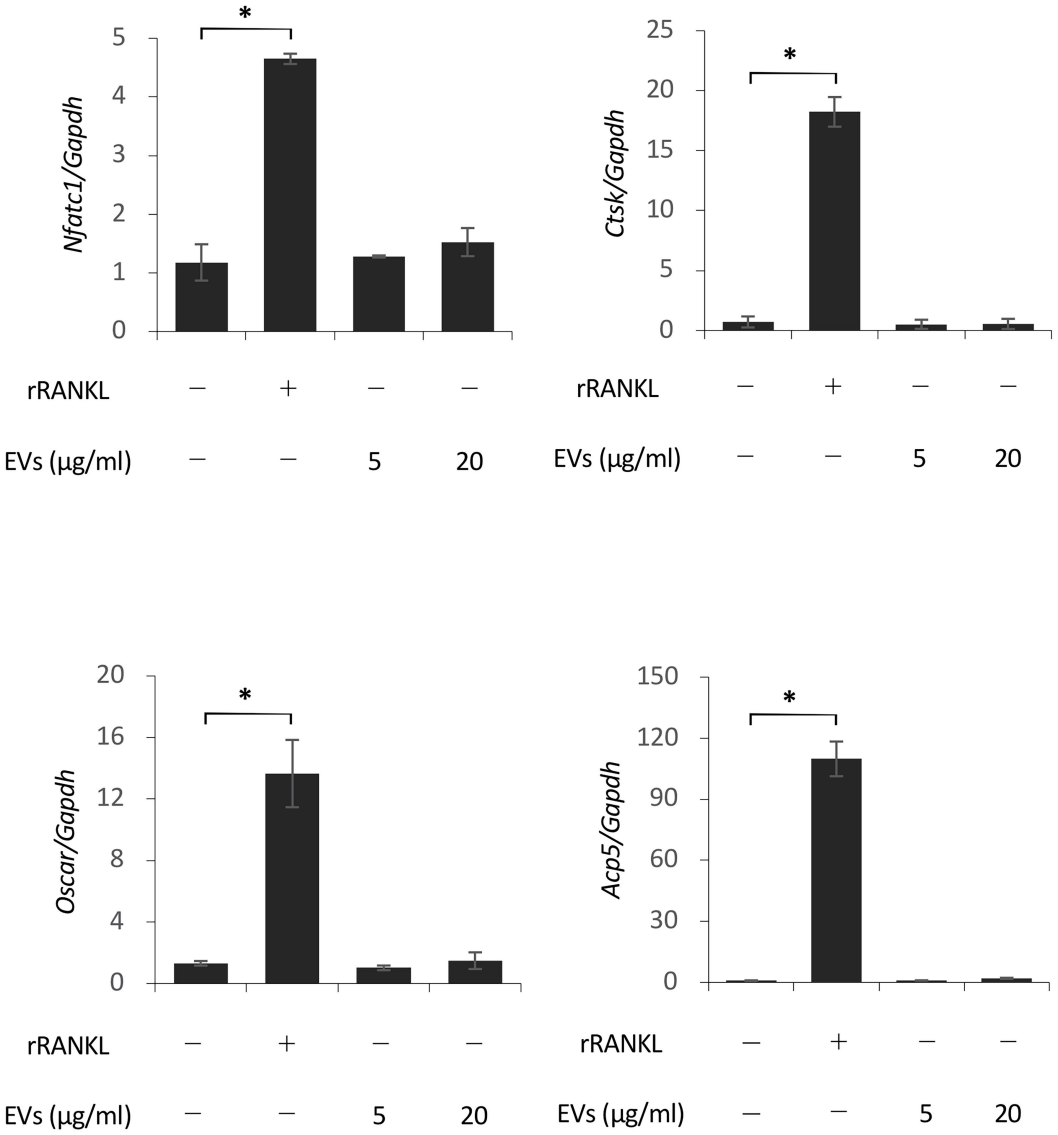
Extracellular vesicles do not induce osteoclast-associated molecules in the absence of rRANKL. RAW 264.7 cells were stimulated with rRANKL (50 ng/ml) or the indicated concentrations of EVs for 3 days. Total cellular RNA was extracted, and the transcripts of *Nfatc1*, *Ctsk*, *Oscar*, and *Acp5* were analyzed by real-time quantitative PCR. Representative findings of three independent experiments are shown (^*^p < 0.05 compared with the control).

We next focused on the molecular mechanism by which EVs enhanced RANKL-induced osteoclast differentiation. RAW 264.7 cells were cultured with rRANKL in the presence or absence of EVs for 3 days, and real-time PCR analysis was conducted to examine the gene expression levels of osteoclast-associated molecules, such as *Nfatc1*, *Ctsk*, *Oscar*, *Acp5* (also known as *Trap*), *Dcstamp*, and *Ocstamp*. As shown in previous reports, rRANKL induced the expression of each of these molecules (Figure 4). The addition of EVs to the rRANKL-treated group resulted in an increased expression of all these genes. Western blot analysis also confirmed that EVs enhanced the protein expression of CtsK and Nfatc1 (Figures 5A,B). On the other hand, when RAW 264.7 cells were stimulated with EVs in the absence of rRANKL, the expression of *Nfatc1*, *Ctsk*, *Oscar*, and *Acp5* did not significantly change compared with control (Figure 6). These findings are consistent with the TRAP staining analysis shown in Figure 3, in which cementoblast EVs alone did not induce osteoclastogenesis.

### RANKL Is Not Expressed by EVs

**Figure 7:**
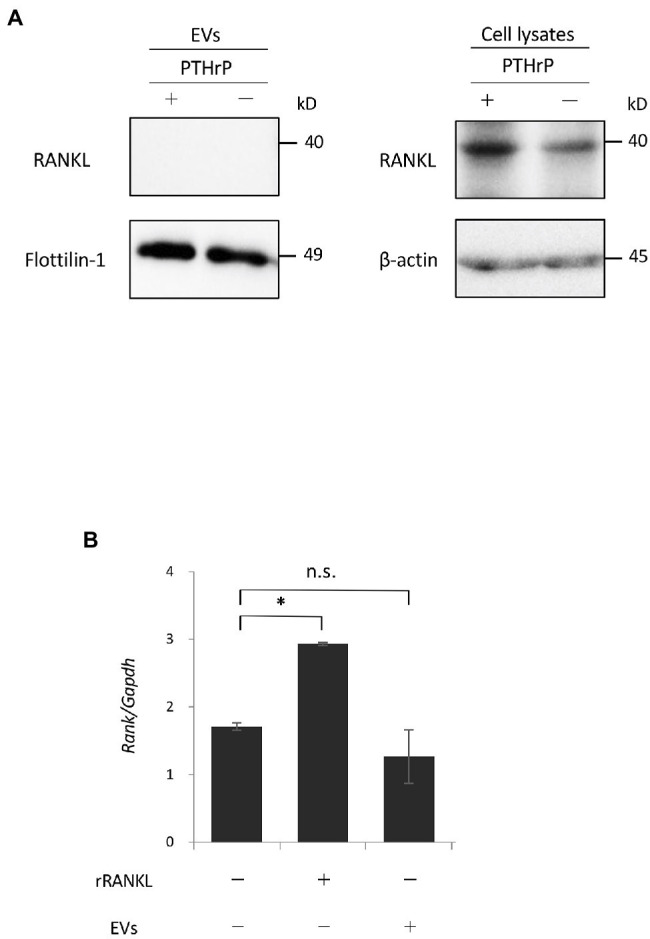
Receptor activator of NF-κB ligand is not expressed by EVs. **(A)**: OCCM-30 cells were stimulated with or without rPTHrP (100 ng/ml) for 48 h, and whole cell lysates or EVs were purified. A Western blot analysis using RANKL antibody was performed using Flottilin-1 and β-actin as reference proteins for EVs and the plasma membrane, respectively. **(B)**: RAW 264.7 cells were stimulated with rRANKL (50 ng/ml) or EVs for 3 days. Total cellular RNA was extracted, and the transcripts of RANK were analyzed by real-time quantitative PCR. Representative findings of three independent experiments are shown (^*^p < 0.05 compared with the control; ns, not significant).

These data suggest that cementoblast-derived EVs may not express RANKL. In previous studies, RANKL was slightly expressed by OCCM-30 cells (Boabaid et al., 2004; Nemoto et al., 2006), and stimulation with PTHrP increased RANKL expression (Boabaid et al., 2004). We analyzed whether RANKL protein was expressed in EVs by a Western blot analysis using PTHrP-stimulated OCCM-30 cells as a positive control. As shown in Figure 7A and previously reported, a certain level of RANKL expression was detected in the cell lysates of unstimulated cultured OCCM-30 cells, and the expression was enhanced by PTHrP stimulation of the cells. However, no RANKL expression was detected in EVs regardless of PTHrP stimulation. Flottilin-1 and β-actin were used as exosomal and cytoplasmic reference proteins, respectively. Real-time PCR analysis revealed that the gene expression of RANK, a receptor for RANKL, in RAW 264.7 cells was not significantly altered upon stimulation with EVs alone (Figure 7B). These findings suggest that the enhancement of osteoclast differentiation by EVs may be due to intracellular signaling downstream of RANK/RANKL binding.

### Conditioned Medium From OCCM-30 Cells Abrogates the EV-Induced Enhancement of Osteoclastogenesis

**Figure 8:**
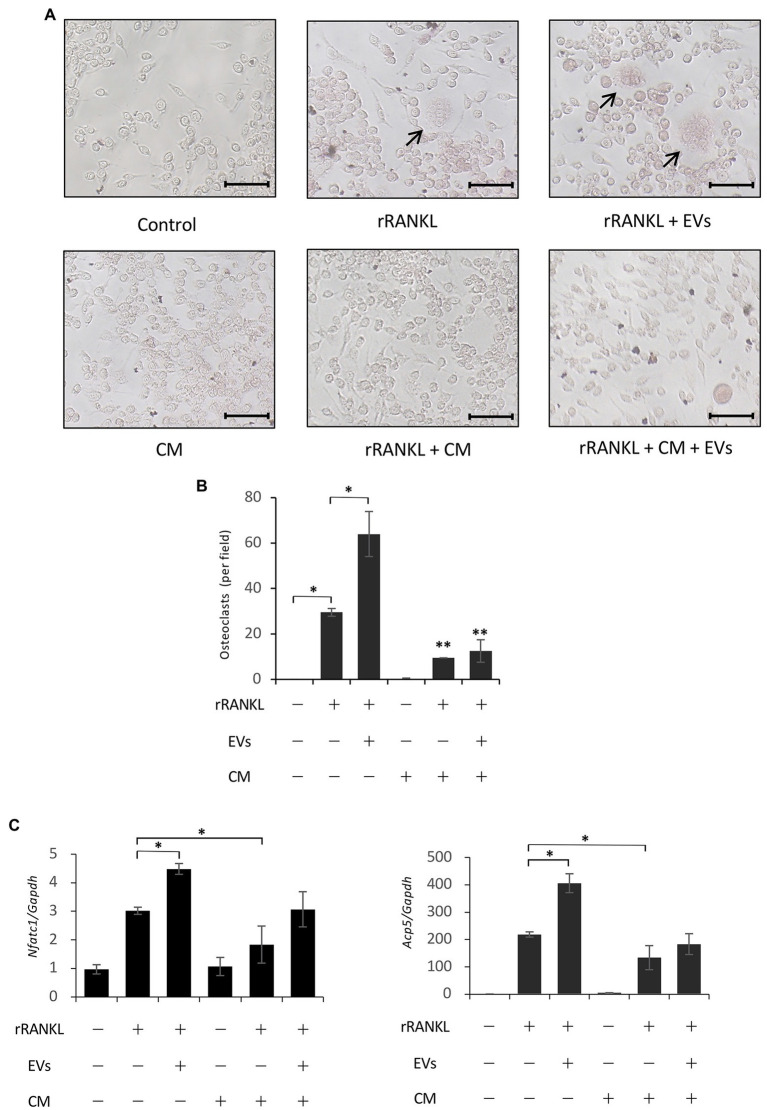
Conditioned medium derived from OCCM-30 cells abrogates EV-mediated enhanced osteoclastogenesis. RAW 264.7 cells were stimulated with rRANKL (50 ng/ml) in the presence or absence of 20 μg/ml EVs or conditioned medium (CM) for 5 days (for osteoclast formation assay) or for 3 days (for gene expression assay). **(A)**: TRAP staining was performed. The TRAP-positive cells were marked with arrows (scale bars: 50 μm). **(B)**: Quantification of the TRAP-positive osteoclasts shown in **(A)**. Results are representative **(A)** or the mean ± SE **(B)** of at least three independent experiments (^*^p < 0.05 compared with the respective control; ^**^p < 0.05 compared with the rRANKL-treated group). **(C)**: Total cellular RNA from RAW 264.7 cells was extracted, and the transcripts of the osteoclast-associated molecules *Nfatc1*, *Ctsk*, *Oscar*, and *Acp5* were analyzed by real-time quantitative PCR. Representative findings of three independent experiments are shown (^*^p < 0.05 compared with the rRANKL-treated group).

As described in the Introduction section, cementum is more poorly absorbed than bone during inflammation. Therefore, we investigated the effects of total soluble factors secreted from cementoblasts, i.e., CM, on RANKL-induced osteoclastogenesis. The CM of cementoblasts contains a variety of soluble factors, such as soluble proteins, free nucleic acids, and lipids, in addition to EVs. RAW 264.7 cells were cultured in rRANKL in the presence of CM, and osteoclastogenesis was measured by TRAP staining after 5 days of culture and the expression of osteoclast-associated genes was assessed by real-time PCR after 3 days of culture. TRAP staining confirmed rRANKL-induced osteoclast differentiation and its enhancement by EVs (Figure 8A, two upper right panels, and Figure 8B). In addition, *Nfatc1* and *Acp5* were expressed (Figure 8C). Next, RAW 264.7 cells were cultured with CM, which theoretically contained about 20 μg/ml of EVs, in the presence of rRANKL (rRANKL + CM group). As shown in Figure 8A (lower middle panel) and Figure 8B, few TRAP-positive multinucleated osteoclasts were detected, and the expression of Nfatc1 and Acp5 was significantly lower than in the rRANKL-treated group (Figure 8C). The addition of EVs to the CM in the presence of rRANKL (rRANKL + CM + EVs group) slightly increased the gene expression of *Nfatc1* and *Acp5* to a level resembling the rRANKL group (Figure 8C), but some TRAP-positive multinucleated osteoclasts were also observed (Figure 8A, right lower panel, and Figure 8B). These results suggest that OCCM-30 cells secrete soluble extracellular factors that partly inhibit RANKL-induced osteoclast differentiation and almost completely inhibit the enhancing effect of EVs.

## Discussion

Cementoblasts play a crucial role in the maintenance of periodontal tissue homeostasis, tissue repair, and regeneration, as well as in the inflammatory response of periodontal tissues, including the activation of the innate immune system *via* Toll-like receptors (Saygin et al., 2000; Bosshardt, 2005; Nemoto et al., 2006). However, interactions between cementoblasts and other cells in periodontal tissues are not well characterized. Here, we found that EVs derived from cementoblasts can facilitate RANKL-mediated osteoclast formation, which may represent a novel mechanism for cementoblast and osteoclast communication.

Bone remodeling is tightly controlled and largely dependent on cellular communication between osteoclasts and osteoblasts through direct cell–cell contact or through the transfer of secreted soluble molecules. Recent studies have suggested that the intercellular transfer of EVs may also act as an important regulator of cell–cell communication (Kulshreshtha et al., 2013; Deng et al., 2015; Lamichhane et al., 2015; Anderson et al., 2016; Cui et al., 2016; Barile et al., 2017; Zhang et al., 2017; Cappariello et al., 2018; Haj-Salem et al., 2018; Kojima et al., 2018; Wang et al., 2018; Jiang et al., 2019; Ni et al., 2019). EVs are enriched with bioactive molecules such as proteins, nucleic acids, and lipids. Among the several means by which EVs exert biological effects are ligand/receptor interactions on target cell membranes (Muller et al., 2017). It has been reported that osteoblast-derived EVs contain RANKL protein at the surface to interact with osteoclast precursors that are specifically recognized through the receptor ligand interaction (RANKL-RANK), resulting in the stimulation of RANKL-RANK signaling to facilitate osteoclast formation (Deng et al., 2015; Cappariello et al., 2018). In the present study, a Western blot analysis revealed that RANKL protein was not detected by cementoblasts-derived EVs. Furthermore, we showed that EVs alone did not induce the osteoclastogenesis of RAW 264.7 cells unless rRANKL was added exogenously and that RANK expression on the cells was not altered in the same condition, suggesting that the enhancement of osteoclastogenesis through EVs may be regulated not at the level of RANK-RANKL binding but in a downstream intracellular signaling pathway.

It has been reported that Wnt5a increases RANK expression on osteoclast progenitor cells through its binding to receptor tyrosine kinase-like orphan receptor-2 (Ror2), thereby promoting osteoclast differentiation (Maeda et al., 2012). Wnt5a has been reported to be expressed in OCCM-30 cells as well as mouse cementoblasts during tooth root formation (Sakisaka et al., 2015). However, given that the RANK gene expression in RAW 264.7 cells was not significantly altered by EVs stimulation, even if EVs express Wnt5a, it is unlikely that Wnt5a is involved in the enhancement of osteoclastogenesis.

Exosomes may be taken up by target cells through several different mechanisms, such as fusion with the plasma membrane, micropinocytosis, phagocytosis, clathrin-mediated, caveolin-dependent, lipid raft-dependent endocytosis, and receptor-mediated endocytosis (Mulcahy et al., 2014). In this experiment, it was shown that EVs was internalized into cells. Once internalized, EVs can fuse their membranes with the plasma membrane of the target cell, resulting in the transfer of various bioactive molecules. Numerous studies suggest that miRNA transfer is an important mechanism for the function of EVs, and miRNAs are involved in the regulation of osteoclast differentiation (Franceschetti et al., 2014). Thus, it is likely that miRNAs are involved in the enhancement of osteoclastogenesis *via* EVs. It has been reported that RANKL-induced osteoclastogenesis is promoted by several miRNAs, such as miR-148a and miR-199a-5p for targeting MAFB (V-maf musculoaponeurotic fibrosarcoma oncogene homolog B; Cheng et al., 2013), miR-214 for targeting the Pten tensin homolog (Zhao et al., 2015), and miR-21 for targeting programmed cell death 4 (Sugatani et al., 2011). Future investigations are required to clarify the genetic information encapsulated in cementoblast-derived EVs in terms of the expression of miRNAs associated with the modulation of osteoclastogenesis.

Cementum is often poorly absorbed in situations such as periodontitis, where osteoclasts absorb alveolar bone. It remains unclear whether this less absorbable property is due to the biochemical composition of the cementum, to the properties of related cells, such as cementoblasts (e.g., possible signaling mechanisms that constitutively inhibit osteoclastogenesis), or to longer distances between the cementum and the vasculature supplying osteoclast precursors to the cementum. It has been demonstrated that the expression of RANKL is low in cementoblasts, but OPG is constitutively synthesized and secreted (Boabaid et al., 2004; Nemoto et al., 2006), which suggests that cementoblasts may be responsible for maintaining lower levels of osteoclastic activity at the root surface. In the present study, although, we showed that EVs have the potential to enhance osteoclastogenesis, the expression of RANKL was not detected in cementoblast-derived EVs, unlike osteoblasts (Deng et al., 2015). Furthermore, cementoblasts secreted soluble factors in CM that partially inhibited RANKL-induced osteoclastogenesis and almost completely abolished the EV-mediated enhancement of osteoclastogenesis. This observation is consistent with cementoblasts playing a protective role against osteoclast resorption. Given that cementoblasts produce OPG at a relatively high level (Boabaid et al., 2004; Nemoto et al., 2006), it is likely that OPG in CM may partially inhibit RANKL-mediated osteoclastogenesis. However, an analysis of OPG-deficient mice revealed that OPG is not the only factor inhibiting osteoclastogenesis in tooth root, as the mice showed no significant abnormalities in cementum but a high degree of bone resorption in alveolar bone (Oshiro et al., 2002). Therefore, cementoblasts may secrete an osteoclastogenesis-inhibitory soluble factor(s) independent of osteoprotegerin (Kim et al., 2008). Such a factor(s) deserves further investigation.

Although cementum is generally considered to be resistant to osteoclast resorption, cementum resorption is often observed in lesions caused by pathological stimuli such as trauma, orthodontic forces, or large periapical periodontitis (Feller et al., 2016). Treating human cementoblasts with IL-1β potently upregulates RANKL expression but not OPG expression, leading to a higher RANKL/OPG ratio and capacity for osteoclastogenesis (Huynh et al., 2017). In several studies using mouse cementoblast cell lines, a higher RANKL/OPG ratio has been shown to occur under the influence of various factors, such as sclerostin (Bao et al., 2013) and prostaglandin E2 (Oka et al., 2007). Moreover, at sites of inflammation, RANKL may be supplied by a variety of cells (Liu et al., 2003; Chen et al., 2014), including T and B lymphocytes, vascular endothelial cells, epithelial cells, and gingival/periodontal fibroblasts, as well as osteoblasts. Our previous experiments suggested cementoblasts recruit osteoclastic precursor cells by inducing chemokines through the activation of toll-like receptor-2 under proinflammatory conditions (Nemoto et al., 2006, 2008). Furthermore, cementoblasts and osteoclast precursor cells have been reported to contact tightly *via* the expression of adhesion molecules, such as vascular cell adhesion molecule-1 (Berry et al., 2008), in pathological processes. Thus, the formation of a microenvironment favoring RANKL-RANK binding may prevent the interaction of OPG with RANKL, which would allow EVs to actively exert their enhancing effect on RANKL-induced osteoclastogenesis. On the other hand, it has been reported that osteoclast formation is induced independently of RANKL (Kim et al., 2005; Feng et al., 2019) through many humoral factors, such as TNF-α, interleukin (IL)-1, IL-6, IL-11, TGF-β, and LPS. Although EVs alone (i.e., without rRANKL) did not induce osteoclast differentiation in the present study, future work should investigate the possibility of the RANK/RANKL/OPG axis-independent osteoclastogenesis by combining EVs with the above humoral factors.

Parathyroid hormone related protein increases RANKL expression in osteoblasts and cementoblasts at the cellular level (Lee and Lorenzo, 1999; Boabaid et al., 2004). In the present study, a similar result was found using OCCM-30 cells. Additionally, RANKL has been reported to be expressed in the EVs of osteoblasts, and its expression is further increased when cells are stimulated with PTHrP (Deng et al., 2015; Cappariello et al., 2018). In the present study, however, RANKL was not expressed by EVs derived from OCCM-30 cells regardless of whether the cells were stimulated with PTH. This observation may partly explain the clinical relevance that cementum often resists resorption under conditions where alveolar bone is targeted by osteoclasts.

In conclusion, these findings not only help us better understand the importance of cementoblast-derived EVs in the physiological and/or pathological biology of periodontal tissues, especially tooth resorption, but also contribute to the therapeutic development of root resorption in pathological processes by exploring the mechanisms that control the osteoclast-enhancing activity of EVs.

## Data Availability Statement

The original contributions presented in the study are included in the article/Supplementary Material, further inquiries can be directed to the corresponding author.

## Author Contributions

RS and EN: study conception and design and writing the manuscript. RS, KM, YS, SS, JL, and KN: acquisition of data. RS, KM, EN, YS, SS, JL, KN, HT, and SY: analysis and interpretation of data. EN: final approval of the article. All authors contributed to the article and approved the submitted version.

## Funding

This study was supported by a Grant-in-Aid for Scientific Research (20K09933).

## Conflict of Interest

The authors declare that the research was conducted in the absence of any commercial or financial relationships that could be construed as a potential conflict of interest.

## Publisher’s Note

All claims expressed in this article are solely those of the authors and do not necessarily represent those of their affiliated organizations, or those of the publisher, the editors and the reviewers. Any product that may be evaluated in this article, or claim that may be made by its manufacturer, is not guaranteed or endorsed by the publisher.
