# Supplementary figures and images for: Extracellular Vesicles Derived From Murine Cementoblasts Possess the Potential to Increase Receptor Activator of Nuclear Factor-κB Ligand-Induced Osteoclastogenesis
Source: Front Physiol. 2022 Feb 14;13:825596. doi: 10.3389/fphys.2022.825596 (PMC8882962; doi:10.3389/fphys.2022.825596)

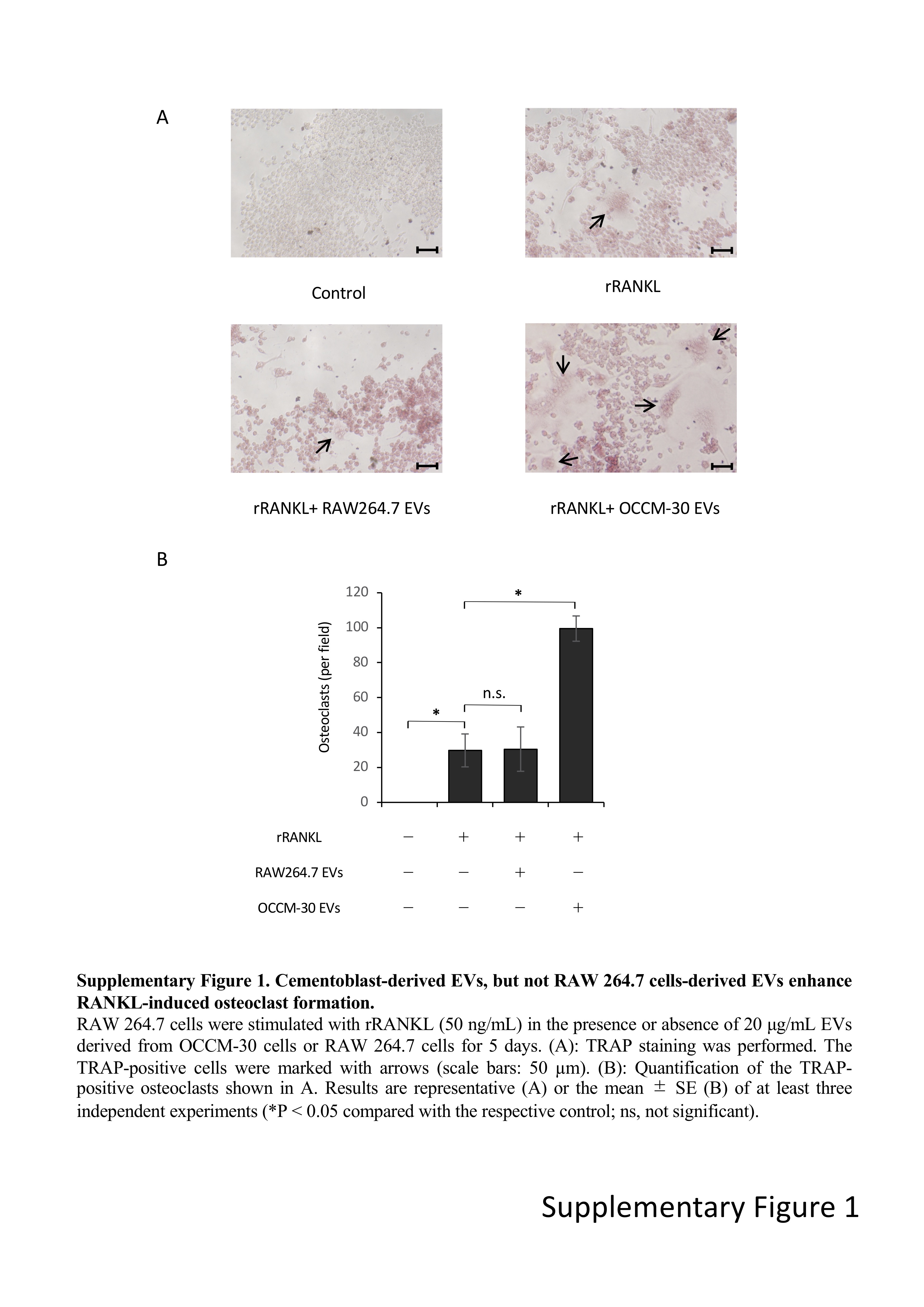

Supplement: Supplementary file 1 [file Image_1.JPEG]
